# Data on a single oral dose of camu camu (*Myrciaria dubia*) pericarp extract on flow-mediated vasodilation and blood pressure in young adult humans

**DOI:** 10.1016/j.dib.2017.12.009

**Published:** 2017-12-14

**Authors:** Tadayoshi Miyashita, Ryosuke Koizumi, Takao Myoda, Yoshimasa Sagane, Koichi Niwa, Toshihiro Watanabe, Kazuhiro Minami

**Affiliations:** a DHC Corporation, 2-7-1 Minami-Azabu, Minato-ku, Tokyo 106-8571, Japan; b NODAI Research Institute, 1-1-1 Sakuragaoka, Setagaya-ku, Tokyo 156-8502, Japan; c Department of Food and Cosmetic Science, Faculty of Bioindustry, Tokyo University of Agriculture, 196 Yasaka, Abashiri 099-2493, Japan

## Abstract

This data article describes the flow-mediated vasodilation (FMD) responses, represented by changes in arterial diameter, and blood pressure changes in young adults after a single oral dose of camu camu (*Myrciaria dubia*) pericarp extract or placebo (cross-over design). Ten healthy men and 10 healthy women participated in this study. Ultrasonic diagnostic equipment was used to monitor arterial diameter changes, indicative of FMD, for 110 s after the administration of the camu camu extract or placebo. In addition, the systolic and diastolic blood pressure values were recorded.

**Specifications Table**

**Table:** 

Subject area	*Biology*
More specific subject area	*Vascular physiology*
Type of data	*Tables and Figures*
How data was acquired	*Flow-mediated vasodilation test using ultrasonic diagnostic equipment*
Data format	*Raw, Analyzed*
Experimental factors	*The brachial artery was monitored using ultrasonic diagnostic equipment, and the diameter was calculated. In addition, blood pressure was assessed.*
Experimental features	*A cross-over design was utilized. The camu camu extract/placebo was administered under conditions of dietary restrictions to avoid confounding.*
Data source location	*Abashiri, Japan*
Data accessibility	*The data are supplied with this article.*

**Value of the data**

• The data provide insights into the effect of camu camu pericarp extract on blood flow-mediated vasodilation and blood pressure.
• The data can be used as a reference for comparisons with other foods that affect flow-mediated vasodilation and blood pressure.
• The data describe a valuable and searchable functional food resource and can be used for future functional food studies.

## 1 Data

**Fig. 1:**
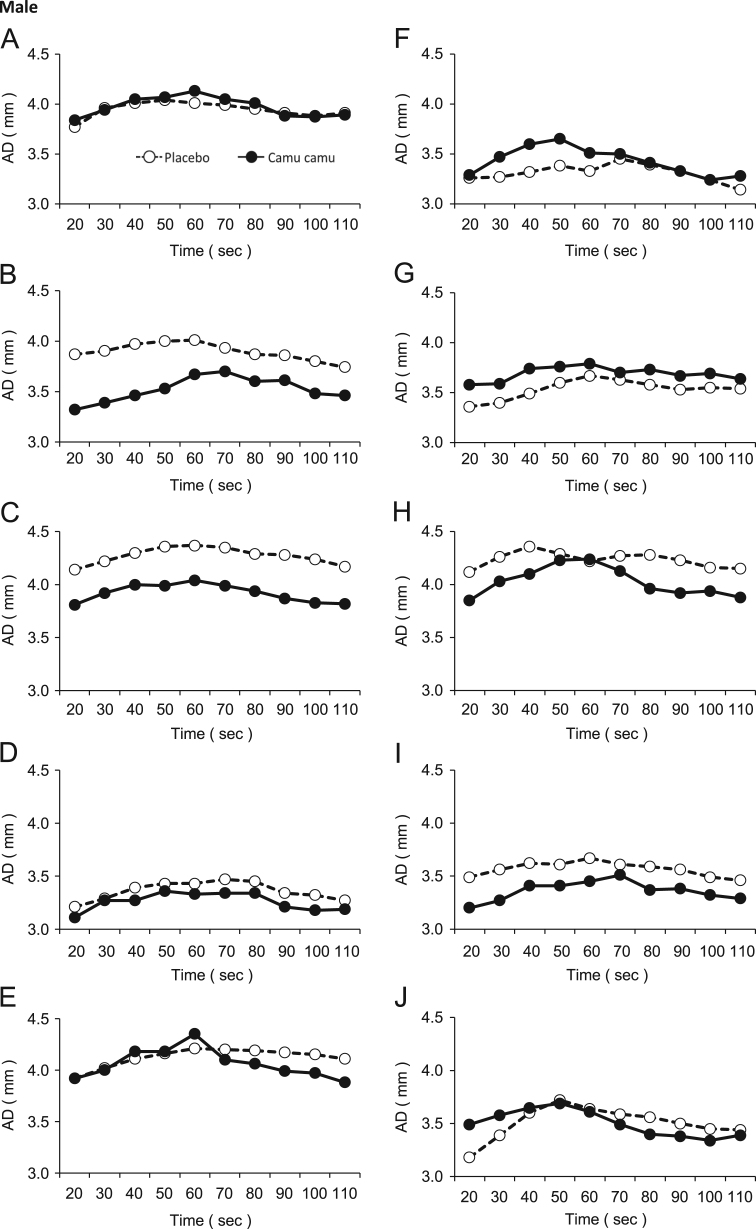
Arterial diameter changes by the FMD response in the male participants administered either the camu camu pericarp extract or placebo.

**Fig. 2:**
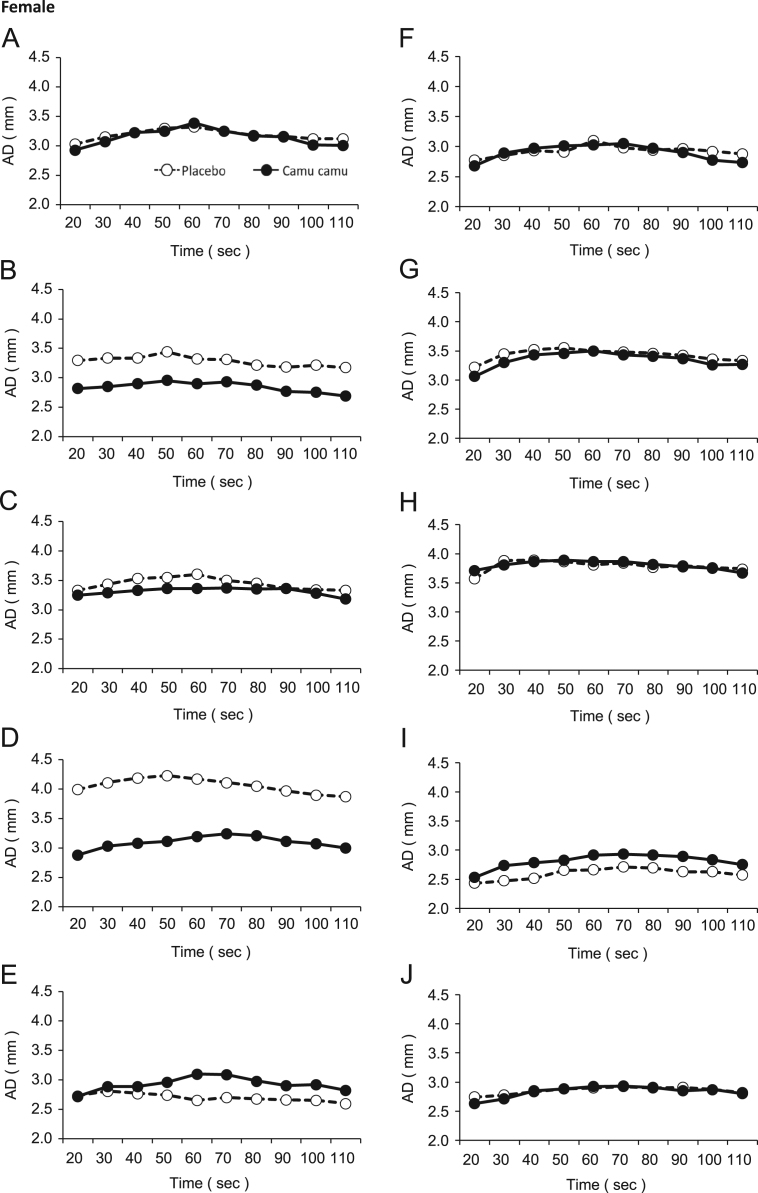
Arterial diameter changes by the FMD response in the female participants administered either the camu camu pericarp extract or placebo.

Flow-mediated vasodilation (FMD), which is the dilation response of the artery to the shear stress of blood flow, can be represented by the changes in brachial arterial diameter after an increase in blood flow. In clinical medicine and functional food studies, FMD measurements serve as a noninvasive method to study endothelial function [1], [2], [3]. The changes in arterial diameter after the administration of camu camu pericarp extract or a placebo are shown in Fig. 1, Fig. 2. The arterial diameter values that were used to generate the line graphs in Fig. 1 are presented in Table 1. The blood pressure data recorded are shown in Table 2.

**Table 1:** Arterial diameter (mm) changes by the FMD response in the participants administered either the camu camu pericarp extract or placebo.

			Time (s)									
			20	30	40	50	60	70	80	90	100	110
Male	A	Placebo	3.77	3.96	4.01	4.04	4.01	3.99	3.95	3.91	3.88	3.91
		Camu camu	3.84	3.94	4.05	4.07	4.13	4.05	4.01	3.88	3.87	3.89
											
	B	Placebo	3.87	3.90	3.97	4.00	4.01	3.93	3.87	3.86	3.80	3.74
		Camu camu	3.32	3.39	3.46	3.53	3.67	3.70	3.60	3.61	3.48	3.46
											
	C	Placebo	4.14	4.22	4.30	4.36	4.37	4.35	4.29	4.28	4.24	4.17
		Camu camu	3.81	3.92	4.00	3.99	4.04	3.99	3.94	3.87	3.83	3.82
											
	D	Placebo	3.21	3.29	3.39	3.43	3.43	3.47	3.45	3.34	3.32	3.27
		Camu camu	3.11	3.27	3.27	3.36	3.33	3.34	3.34	3.21	3.18	3.19
											
	E	Placebo	3.92	4.02	4.11	4.16	4.21	4.20	4.19	4.17	4.15	4.11
		Camu camu	3.92	4.00	4.18	4.18	4.35	4.10	4.06	3.99	3.97	3.88
											
	F	Placebo	3.26	3.27	3.32	3.38	3.33	3.45	3.39	3.33	3.24	3.14
		Camu camu	3.29	3.47	3.60	3.65	3.51	3.50	3.41	3.33	3.24	3.28
											
	G	Placebo	3.36	3.40	3.49	3.60	3.67	3.63	3.58	3.53	3.55	3.54
		Camu camu	3.58	3.59	3.74	3.76	3.79	3.70	3.73	3.67	3.69	3.64
											
	H	Placebo	4.12	4.26	4.36	4.29	4.22	4.27	4.28	4.23	4.16	4.15
		Camu camu	3.85	4.03	4.10	4.23	4.24	4.13	3.96	3.92	3.94	3.88
											
	I	Placebo	3.49	3.56	3.62	3.61	3.67	3.61	3.59	3.56	3.49	3.46
		Camu camu	3.20	3.27	3.41	3.41	3.45	3.51	3.37	3.38	3.32	3.29
											
	J	Placebo	3.18	3.39	3.60	3.72	3.64	3.59	3.56	3.50	3.45	3.44
		Camu camu	3.49	3.58	3.65	3.69	3.61	3.49	3.40	3.38	3.34	3.39
												
Female	A	Placebo	3.03	3.15	3.23	3.30	3.32	3.25	3.18	3.16	3.12	3.12
		Camu camu	2.93	3.07	3.23	3.25	3.39	3.25	3.17	3.15	3.02	3.01
											
	B	Placebo	3.29	3.33	3.33	3.44	3.32	3.31	3.21	3.18	3.21	3.17
		Camu camu	2.82	2.85	2.90	2.95	2.90	2.93	2.87	2.77	2.75	2.69
											
	C	Placebo	3.33	3.43	3.53	3.55	3.60	3.50	3.45	3.36	3.34	3.33
		Camu camu	3.25	3.29	3.33	3.36	3.36	3.37	3.35	3.36	3.28	3.18
											
	D	Placebo	3.99	4.11	4.19	4.23	4.17	4.11	4.05	3.97	3.90	3.87
		Camu camu	2.88	3.03	3.08	3.11	3.19	3.24	3.21	3.11	3.07	3.00
											
	E	Placebo	2.73	2.81	2.77	2.74	2.65	2.70	2.68	2.66	2.65	2.60
		Camu camu	2.72	2.89	2.89	2.96	3.10	3.09	2.98	2.90	2.92	2.82
											
	F	Placebo	2.77	2.85	2.93	2.91	3.10	2.98	2.94	2.96	2.92	2.88
		Camu camu	2.68	2.89	2.97	3.01	3.03	3.05	2.97	2.90	2.77	2.73
											
	G	Placebo	3.22	3.45	3.52	3.55	3.50	3.48	3.46	3.42	3.36	3.33
		Camu camu	3.06	3.30	3.43	3.46	3.50	3.43	3.41	3.37	3.26	3.27
											
	H	Placebo	3.57	3.88	3.89	3.87	3.81	3.84	3.77	3.79	3.76	3.74
		Camu camu	3.71	3.81	3.87	3.89	3.87	3.87	3.82	3.78	3.75	3.67
											
	I	Placebo	2.43	2.47	2.51	2.65	2.66	2.71	2.69	2.63	2.63	2.57
		Camu camu	2.53	2.73	2.78	2.82	2.91	2.93	2.91	2.89	2.83	2.75
											
	J	Placebo	2.74	2.78	2.83	2.88	2.90	2.92	2.90	2.91	2.87	2.82
		Camu camu	2.63	2.71	2.85	2.88	2.92	2.93	2.91	2.85	2.87	2.80

**Table 2:** Blood pressure measurements of the participants administered either the camu camu extract or placebo.

		Blood pressure (mmHg)			
		Systolic		Diastolic	
	Participant	Placebo	Camu camu	Placebo	Camu camu
Male	A	136	133	46	55
	B	128	109	66	64
	C	110	103	53	59
	D	116	99	69	69
	E	118	105	68	55
	F	118	117	68	65
	G	107	103	61	60
	H	113	111	85	73
	I	102	107	67	67
	J	110	111	75	74
					
Female	A	92	97	56	58
	B	89	87	60	54
	C	93	108	55	68
	D	116	102	69	64
	E	61	80	55	46
	F	107	94	70	55
	G	96	80	54	47
	H	94	95	57	61
	I	90	99	57	55
	J	98	98	60	68

## 2 Experimental design, materials and methods

### 2.1 Participants

Twenty healthy students (10 men and 10 women; age range, 18–28 years) from the Hokkaido Okhotsk Campus of the Tokyo University of Agriculture were recruited in July 2017. Individuals with a history of hypertension, diabetes mellitus, or dyslipidemia were excluded because of the potential effects of these conditions on endothelial function. The study protocol was approved by the Tokyo University of Agriculture Committee of Human Subject Research Ethics. Informed consent was obtained from all participants.

### 2.2 Materials

The extract was prepared by stirring dried camu camu pericarps (1.5 kg) in two-fold volume of 50% ethanol for 2 h. The solution was filtered using filter paper, and the filtrate was collected. The filtered residue was subjected to a second round of ethanol extraction with same volume of 50% ethanol. The filtrates from the first and second rounds of ethanol extraction were pooled, and the ethanol was removed by evaporation; the filtrate was then freeze-dried. The freeze-dried powder was encapsulated into soft gelatin capsules (25 mg in each capsule) (Matsuya, Osaka, Japan). Three capsules containing the camu camu extract comprised a single dose; whereas, three soft capsules containing 25 mg of water each were administered as the placebo. The food restriction protocol involved the use of cereal (Flugra, Calbee, Tokyo, Japan) for meals.

### 2.3 Food restriction protocol and FMD measurement

The data were collected at the Hokkaido Okhotsk Campus of the Tokyo University of Agriculture between August and September 2017. By applying a cross-over design, each participant was randomly administered a single dose of the camu camu pericarp extract or water (placebo) in a soft capsule. The second dose was administered more than a week later. The food restriction protocol and FMD were assessed using ultrasonic diagnostic equipment (LOGIQ P6, GE Healthcare, Little Chalfont, UK), as previously reported [1]. Arterial diameter was calculated using the FMDscope software (Media Cross Co. Ltd, Japan). Blood pressure was measured using an electronic sphygmomanometer (ES-P2000, Terumo, Tokyo, Japan).
