# Supplementary material for: Data on a single oral dose of camu camu (Myrciaria dubia) pericarp extract on flow-mediated vasodilation and blood pressure in young adult humans
Source: Data Brief. 2017 Dec 14;16:993–9. doi: 10.1016/j.dib.2017.12.009 (PMC5752086; doi:10.1016/j.dib.2017.12.009)
Supplement: Supplementary file 2 — Supplementary material [file mmc2.pdf]

Certification

Research protocol approval by the Institutional Review Board (IRB)

The IRB approved the following research protocol.

Name	Kazuhiro Minami
Title	Professor
Faculty	Faculty of Bioindustry
Research title	**Study on the effects of cam-cam peel extract on energy metabolism and vascular function**
Identifying number	1714

Hiroharu Kamioka, Prof., Ph.D.

Date: October 18, 2017

Hiroharu Kamioka

Review-chief of the Institutional Review Board,
Tokyo University of Agriculture
